# Accounting for spatial sampling patterns in Bayesian phylogeography

**DOI:** 10.1073/pnas.2105273118

**Published:** 2021-12-20

**Authors:** Stéphane Guindon, Nicola De Maio

**Affiliations:** ^a^Department of Computer Science, Laboratoire d’Informatique, de Robotique et de Microélectronique de Montpellier, CNRS and Université de Montpellier, 34095 Montpellier, France;; ^b^European Molecular Biology Laboratory, European Bioinformatics Institute, Hinxton CB10 1SD, United Kingdom

**Keywords:** phylogeography, statistical modeling, West Nile virus, Bayesian inference, sampling design

## Abstract

Statistical phylogeography provides useful tools to characterize and quantify the spread of organisms during the course of evolution. Analyzing georeferenced genetic data often relies on the assumption that samples are preferentially collected in densely populated areas of the habitat. Deviation from this assumption negatively impacts the inference of the spatial and demographic dynamics. This issue is pervasive in phylogeography. It affects analyses that approximate the habitat as a set of discrete demes as well as those that treat it as a continuum. The present study introduces a Bayesian modeling approach that explicitly accommodates for spatial sampling strategies. An original inference technique, based on recent advances in statistical computing, is then described that is most suited to modeling data where sequences are preferentially collected at certain locations, independently of the outcome of the evolutionary process. The analysis of georeferenced genetic sequences from the West Nile virus in North America along with simulated data shows how assumptions about spatial sampling may impact our understanding of the forces shaping biodiversity across time and space.

## Significance

Statistical phylogeography has led to substantial progress in our understanding of the pace and means by which organisms colonize their habitats. Yet, inference from these models often relies on implicit assumptions pertaining to spatial sampling design, potentially leading to biased estimation of key biological parameters. While sampled locations sometimes convey signal about the processes that shape spatial biodiversity, they do not always do so. We present a statistical approach that permits accurate estimation of dispersal rates, even in cases where spatial sampling is driven by practical motivations unrelated to the outcome of the evolutionary process. The proposed framework paves the way to further developments in phylogeography with key applications, including the efficient monitoring of pandemics and invasive species during the course of their evolution.

The combined analysis of genetic and spatial information provides powerful tools to decipher how evolutionary processes unfold in space and time. Genetic sequences indeed reveal the evolutionary relationships between sampled lineages. Evolutionary distances may in turn be expressed in terms of calendar time units when information is available about the rate at which substitutions or mutations take place or when sequences were sampled at distinct points in time (1). The analysis of spatial coordinates through the lens of evolution then permits the estimation of the rate at which lineages travel in space and the history of their diffusion through space.

Modeling spatial and genetic information in a unified mathematical framework has a long history. The isolation-by-distance model was first proposed in the middle of the 20th century. Because of its fundamental inconsistencies (2), this model was supplanted by a series of “migration-matrix” approaches that approximate the habitat as a set of discrete locations rather than as a continuum (3–8). None of the inference techniques based on these models explicitly accommodate for potential patterns in sampling locations (see ref. 9 for a review) even though the product of dispersal and population density is underestimated (respectively overestimated) if sampled demes are close to (respectively far from) one another (10). These results suggest that sampling patterns, when ignored, could be responsible for biases in the inference.

Spatial sampling issues are difficult to deal with partly because the migration-matrix models rely on a forward-in-time description of the whole population. The structured coalescent (11–13) follows instead a sample of lineages backward in time, thereby naturally accounting for spatial sampling considerations. Still, the occurrence of “ghost demes,” i.e., demes that were not sampled, may bias the inference of both migration and effective subpopulation size parameters (14, 15). Considering the habitat as a continuum instead of discrete demes potentially alleviates some of these issues, although the approach implemented so far is computationally demanding (16).

Properly accommodating for sampling patterns in phylogeography became more prominent in the last decade with the increased availability of georeferenced genetic data combined with the gain in popularity of efficient implementations of Bayesian samplers under new phylogeography models (17, 18). The “mugration” model (19), whereby forward-in-time migration between discrete demes is modeled as a continuous-time Markov chain, is now used extensively for modeling rapidly evolving infectious diseases (20). However, De Maio et al. (21) showed that nonuniform sampling of individuals across the habitat may hamper migration parameter estimation under this model.

Diffusion models represent a useful addition to the arsenal of phylogeographic models since they apply to the cases where the habitat is a continuum. The “relaxed random walk” (RRW) approach (22), in particular, was used in many instances for studying infectious disease outbreaks in humans (see, e.g., ref. 23 for a review). This approach may also be deployed at deeper timescales (24). For instance, the RRW model is considered one of the key tools to reconstruct the spatiotemporal dynamics of species and populations in and out of climate refugia (25). It describes the spatial dynamics of lineages as a Brownian diffusion process running forward in time along a gene genealogy or phylogeny inferred from the genetic sequences at hand. This probabilistic framework, implemented in a Bayesian setting, allows for great flexibility in modeling a variety of phenomena at different evolutionary timescales.

Recently, however, Kalkauskas et al. (26) have shown that patterns in spatial sampling impact the inference under the RRW model in a manner similar to that observed with the mugration model. The biases in parameter estimates result from the implicit assumption that spatial sampling mirrors the outcome of the evolutionary process; i.e., sampling intensity reflects population density. In practice, sampling is generally influenced by practical considerations and some areas of the habitat may be easier to access (e.g., valleys vs. mountainous areas) (27). Many other aspects including socioeconomic factors (wealthy regions are arguably more likely to generate a large volume of data compared to poor ones) may also be responsible for spatial variation in sampling intensity. Spatial sampling is thus a complex and central issue in different research areas related to evolution and ecology. It is in fact listed as the first challenge in phylodynamic inference (28–30). While the present study focuses on sampling issues pertaining to the RRW model, these problems affect all statistical phylogeography methods that rely on a forward-in-time description of the spatiotemporal processes, regardless of the evolutionary timescale considered or the organisms under scrutiny.

Preferential sampling takes place whenever the process generating the data (the sequences and their spatial locations in our case) and that characterizing the sampling process (governing the timing of sequence collection along with the sampled locations) are stochastically dependent. This phenomenon was first formalized in the context of geostatistical inference (31). In population genetics, the impact of preferential sampling on the inference of effective population size has been investigated recently (32–34). Karcher et al. (33) showed that the estimation of that quantity may be systematically biased when sampling times depend on the dynamics of the population’s demography and the inference is ignorant of that information.

In statistical phylogeography, preferential sampling may take place when the probability of collecting a sample at a particular location in space depends on the likelihood with which the evolutionary process generated sequences in that area. Here again, preferential sampling needs to be explicitly accounted for when modeling the processes that delineate the sampled areas (and the timing of sequence collection) as well as the mechanisms that generate the sequences and their locations. For example, when considering the severe acute respiratory syndrome coronavirus 2 (SARS-CoV-2) pandemic, one of the most important issues with respect to reconstructing the dynamics of geographical spread of the virus has been the fact that some countries have sequenced many more SARS-CoV-2 genomes than others. This biased representation of different geographic locations in genomic datasets affects the inference methods (30) and motivated the development of new, ad hoc approaches (35). In a different context, biases in spatial sampling are a confounding factor that explains the positive association between human density and the diversity of amphibians and reptiles in Europe (36). Spatial sampling considerations therefore go beyond the sole analysis of viral pathogens and properly taking them into account is also paramount to designing adequate techniques for understanding the ecological processes shaping biodiversity.

The present study addresses issues pertaining to preferential sampling in Bayesian phylogeography under the RRW model (22). We introduce a mathematical framework that accommodates for two distinct sampling schemes. The first scheme, referred to as the detection scheme, corresponds to the situation where spatial sampling is either complete or proportional to the underlying population density. The second sampling scheme, which we call the survey scheme, applies to the common situation where some areas are easier to get samples from compared to other regions of the habitat. Importantly, inference under this last scheme does not require modeling the variations in the intensity with which various regions of the habitat may be sampled, although this information may be incorporated in the model. We address the second scenario using the exchange algorithm, a sampling technique that generates random draws from doubly intractable distributions (37, 38).

The analysis of simulated data shows that the biases in dispersal rates that affect phylogeography studies (26) diminish substantially when the inference accommodates for the adequate spatial sampling scheme. The reconstruction of spatiotemporal and demographic dynamics of the West Nile virus in North America further reveals the strong impact that spatial sampling has on phylogeographic inference. Our results indicate that distinct narratives about the spatial dynamics and the demographics of populations or species may derive from the analysis of georeferenced genetic sequences, depending on the sampling scheme considered. The statistical modeling techniques introduced in this study produce a finer picture of the forces governing biodiversity in time and space, thereby providing a solution to central issues in the analysis of georeferenced genetic data.

## Results

**Fig. 1.**
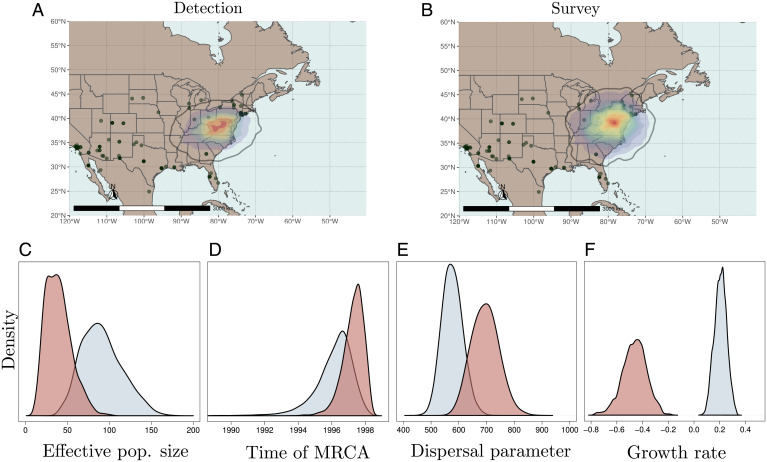
Analyses of WNV data under the detection and survey sampling schemes. (*A*) Estimated location of the MRCA obtained under the detection scheme. (*B*) Estimated location of the MRCA obtained under the survey scheme. The black density line delineates the 95% credibility interval for this parameter. Solid and shaded dots on the maps correspond to the sampled locations. (*C*) Posterior densities of Kingman’s coalescent effective population sizes. (*D*) The age of the root node. (*E*) The dispersal distance per year (in kilometers). (*F*) The exponential growth parameter. Distributions in blue and red were obtained under the detection and survey schemes, respectively.

We compared two spatial sampling schemes: the survey scheme that considers that sampled locations do not convey information about the evolutionary process and the detection scheme that rests on the opposite hypothesis. Fig. 1 shows the posterior densities for parameters of interest obtained from the analysis of the West Nile virus (WNV) dataset analyzed under both sampling schemes. The survey and detection schemes agree that the inferred origin of the WNV epidemic in North America is located in the northeast regions of the United States. Its precise location is less certain under the survey scheme (Fig. 1*B*) compared to the detection scheme (Fig. 1*A*), in particular with regard to the latitudinal component. The effective population size parameter is smaller under the survey scheme (Fig. 1*C*), most likely explaining the more recent estimates for the age of the most recent common ancestor (MRCA) under that scheme (Fig. 1*D*). The signal conveyed by the data about this parameter (as well as the exponential growth parameter) is weak though as its posterior distribution is heavily influenced by the prior (*SI Appendix*, section 3). While dispersal parameter estimates are similar to that obtained in previous studies (39, 40), values of that parameter inferred under the survey scheme are noticeably larger than under the detection scheme, thereby suggesting long dispersal events in short time frames (Fig. 1*E*). Conversely, small dispersal rates favor deep coalescence events, particularly between tip lineages. This observation may explain why the estimates of the population growth parameter obtained under the detection scheme are larger than those derived with the survey scheme (Fig. 1*F*).

We next conducted simulations to assess the precision and accuracy of the inference under the two sampling schemes in cases where samples are collected under various spatial patterns. These patterns, referred to as sampling designs, correspond to various strategies for collecting sequences throughout the habitat. Table 1 gives the 95% highest posterior density (HPD) intervals of dispersal rate estimates and the proportion of simulated datasets where the true rate lies within this interval (see *SI Appendix*, section 4 for the full posterior distributions). For each of the seven sampling designs (*Materials and Methods*), the 95% HPD intervals for the dispersal rate along the *y* axis and the *x* axis are given for the two sampling schemes (detection and survey). Cases where data points are collected around clusters (designs 2 and 6 in Table 1) or on one side of the habitat (designs 4 and 5 in Table 1) demonstrate the impact of spatial sampling on the inference. While estimates of the longitudinal component of the dispersal parameter are mildly or strongly biased under the detection scheme for sampling designs 2 to 6 in Table 1, inference is generally more accurate under the survey scheme overall, with the most noticeable improvements observed for “clustered sampling” designs 2 and 6 as well as for the “identity line” sampling design 3. Additionally, dispersal estimates obtained under the “uniform” (design 1) and the “overdispersed” (design 7) sampling are less accurate under the survey scheme compared to the detection scheme. This observation suggests that this sampling scheme is more sensitive to the prior distribution that applies to these parameters. Additional analyses with a dispersal prior mean set to 2.0 (instead of 10.0) show an increased accuracy and precision under the survey scheme while the quality of the inference remains essentially unchanged under the detection scheme (*SI Appendix*, section 4), illustrating the contrasting impact that prior distributions have, depending on the sampling scheme considered.

**Table 1.** Accuracy and precision of dispersal rate estimates under seven spatial sampling designs: comparison of the detection and survey schemes

Design Scheme		Detection		Survey	
		$\left[q_{0.025},q_{0.975}\right]$	% correct	$\left[q_{0.025},q_{0.975}\right]$	% correct
	Lat.	[0.78, 1.80]	0.90	[0.94, 3.32]	0.65
1) 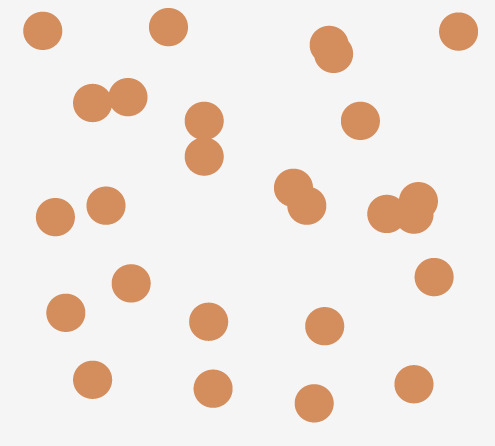	Lon.	[0.75, 1.72]	0.90	[0.92, 3.34]	0.68
	Lat.	[0.11, 0.22]	0	[0.27, 2.32]	0.43
2) 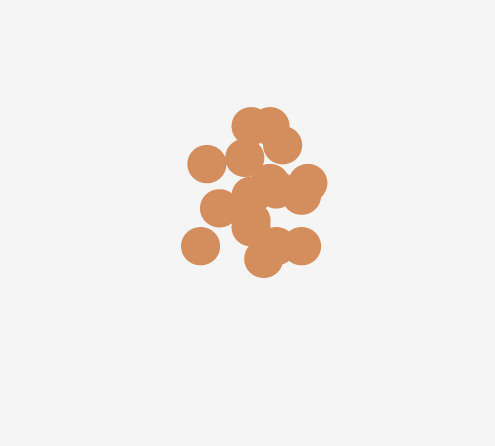	Lon.	[0.12, 0.24]	0	[0.28, 2.93]	0.53
	Lat.	[0.38, 0.87]	0.30	[0.78, 14.64]	0.80
3) 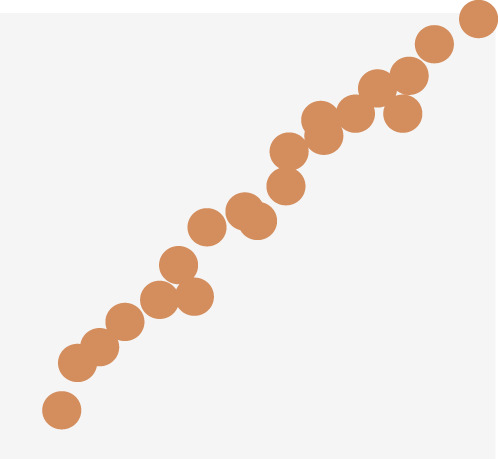	Lon.	[0.39, 0.88]	0.28	[0.76, 12.30]	0.83
	Lat.	[0.74, 1.72]	0.93	[0.83, 2.49]	0.78
4) 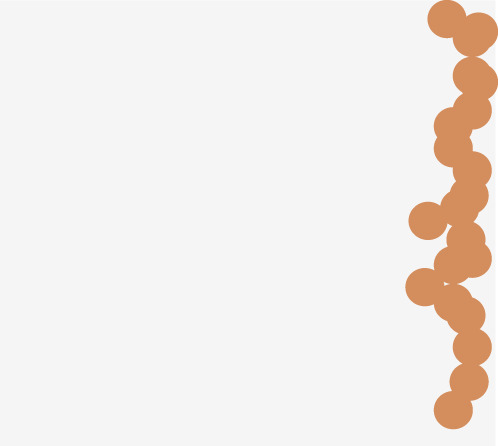	Lon.	[0.27, 0.65]	0.13	[0.59, 4.75]	0.90
	Lat.	[0.74, 1.72]	0.90	[0.87, 3.02]	0.78
5) 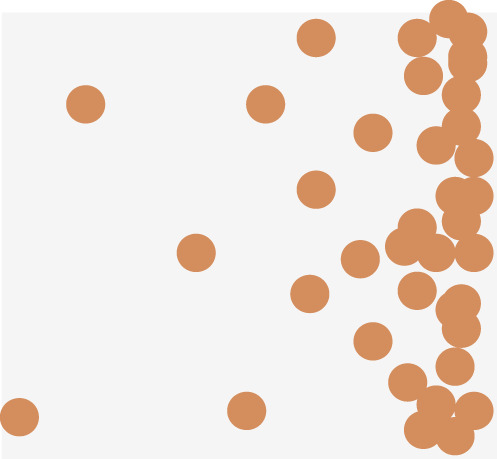	Lon.	[0.54, 1.25]	0.75	[0.67, 2.50]	0.90
	Lat.	[0.10, 0.18]	0	[0.16, 1.09]	0.23
6) 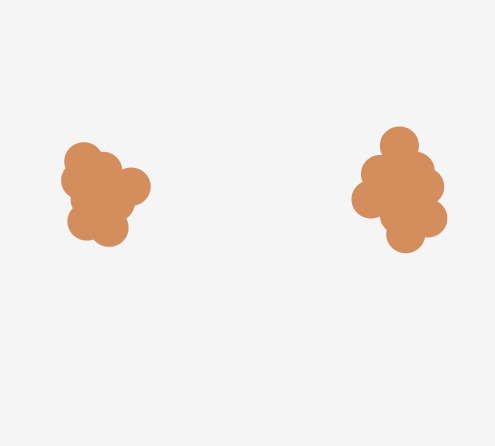	Lon.	[0.47, 1.05]	0.63	[0.97, 4.20]	0.55
	Lat.	[0.77, 1.76]	0.88	[0.93, 3.48]	0.65
7) 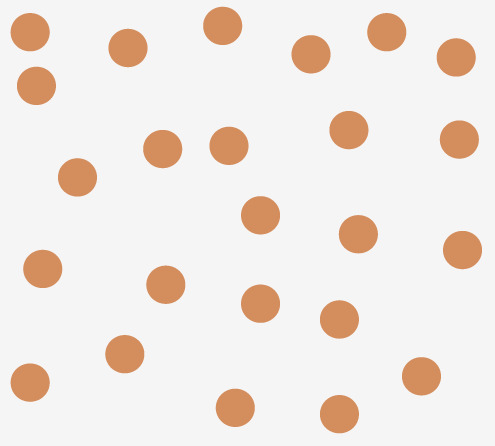	Lon.	[0.79, 1.79]	0.90	[0.92, 3.16]	0.68

“$q_{0.025}$” (respectively “$q_{0.975}$”) is the average taken over 40 simulation replicates of the 0.025 (respectively 0.975) quantile of the posterior distribution for the corresponding dispersal parameter. The “% correct” gives the proportion of simulated datasets where the 95% HPD brackets 1.0, the true value of the dispersal parameters.

Because the exchange algorithm relies on an “inner” Markov chain Monte Carlo (MCMC) sampler (Eq. 8) within a standard “outer” MCMC sampler, inference under the survey scheme is necessarily slower than under the detection scheme. We focused here on the median duration (in seconds) of one iteration of the outer sampler. For the WNV dataset, inference under the survey scheme is ∼40% slower than under the detection scheme with the median duration of one cycle in the MCMC close to 1.4 $\times 10^{-3}$ s under the survey scheme and 1.0 $\times 10^{-3}$ s under the detection scheme. For the simulated datasets, one MCMC step took on average 8.5 .. s and 1.3 $\times 10^{-3}$ s, amounting to ∼ 23 h and ∼ 36 h for completing each dataset analysis under the detection and survey schemes, respectively.

## Discussion

The present study shows that dispersal and demographic parameter inference under popular Bayesian inference models in statistical phylogeography may be substantially affected by the data collection procedure. The standard approach assumes that the spatial density of samples is proportional to that of the underlying population. In some situations, however, sampled locations are selected based on criteria that have little to do with the evolutionary process. This distinction is crucial as it leads to very different probabilistic modeling approaches.

In this study, we explicitly incorporate the sampling strategy in the building of the statistical model. On the one hand, we show that the standard approach, the so-called detection sampling scheme, amounts to considering that the data collection process is fully guided by the outcome of the evolutionary process. On the other hand, we introduce the survey sampling scheme whereby the sampling locations are independent from the evolutionary process. This last scheme is for instance particularly relevant in cases where sampled areas are chosen based on the ease with which these areas can be accessed. Bayesian inference under the survey scheme requires the deployment of a specific sampling technique, the exchange algorithm, that deals with doubly intractable problems. We employ an extension of this algorithm that relies on a Metropolis–Hastings sampler of an auxiliary phylogeography model that runs within the standard sampling algorithm.

The analysis of the West Nile virus dataset shows that parameter estimates can vary substantially depending on the sampling scheme considered. In particular, uncertainty around the location of the MRCA, which is of particular interest when searching for the geographic origins of an epidemic, is larger under the survey scheme. The sampling scheme also impacts demographic parameters that characterize the dynamics of the effective size of the underlying population. Our results suggest that population size is growing under the detection scheme while it is shrinking under the survey scheme. Records of WNV-related disease cases in the United States between 1999 and 2009 (https://www.cdc.gov/westnile/statsmaps/cumMapsData.html) do not clearly support either hypothesis. This result illustrates the necessity to accommodate for various sampling schemes when performing phylogeographic analyses in order to have a nuanced and comprehensive view of the underlying evolutionary processes. It is not clear whether one sampling scheme is more relevant than the other in this particular case. The detection scheme is likely to be more pertinent in the early stages of the epidemic where most if not all new cases were reported and sequenced. The survey scheme is more suitable to the analysis of subsequent stages of the epidemic where the virus occupies the whole habitat and only a fraction of all cases are sampled. In any case, because of its substantial impact on parameter inference, our results demonstrate that any phylogeography analysis should rest on a sound understanding and modeling of the sampling process that generated the data at hand.

The analysis of simulated datasets provides a broad overview of the relative performance of the two sampling schemes under various sampling designs that aim at reproducing the constraints of field surveys. The comparison between detection and survey schemes shows that estimates derived under the survey scheme are generally less precise but more accurate than those obtained with the detection scheme. The diminished precision under the survey scheme is expected as, according to this sampling scheme, sampling locations are not considered as data generated solely by the evolutionary process. For the same reason, dispersal estimates derived under the survey scheme are more dependent on the specifics of the prior distribution, emphasizing the importance to perform sensitivity analyses when conducting Bayesian phylogeography inference studies.

Selecting a sensible spatial sampling scheme prior to analyzing georeferenced genetic data requires careful scrutiny of the experimental design put in place. For instance, field surveys in ecology may rely on sampling designs ranging from comprehensive surveys where the detection scheme is more relevant to very constrained ones where the survey scheme will be more appropriate. Combining these two sampling schemes in the same analysis could also be relevant to cases where sampling is comprehensive early on and partial in subsequent stages of evolution, as could be the case for viral pandemics. Also, the present study deals with a continuous diffusion model to describe the movement of lineages during the course of evolution. Combining various spatial sampling schemes to cases where the population or species of interest is structured into discrete demes is another potential extension of our work. Doing so would in fact make the mugration model more readily comparable to the standard structured coalescent model. Finally, the location of sampled lineages is often known with limited precision in practice. Extending previous work (41), Dellicour et al. (40) recently proposed a generalization of the standard model whereby lineages may be found within polygons of various areas. Combining this approach with the techniques presented in the present study would considerably enhance the set of tools to deal with spatial sampling designs in statistical phylogeography.

Although phylogeography and phylodynamics hold great promise for understanding the evolutionary mechanisms that govern the spatial distribution of related organisms, proper statistical modeling of the underlying processes is paramount. While substantial progress has been made over the years in modeling the stochastic processes unfolding along the evolutionary trees, accommodating for realistic sampling designs remains a vast and largely untackled issue. The present study shows how important it is to explicitly incorporate spatial sampling in the inference of probabilistic models in phylogeography and paves the way to further developments in this area.

## Materials and Methods

### Notation.

In the following, we use p(·) for a generic probability density. The density f(·) corresponds to that defined by the forward-in-time Brownian diffusion process governing the spatial component of the phylogeographic model. The present study rests on the RRW model introduced in ref. 22. This model describes the location of lineages during the course of evolution with $l^{*}$ the vector of locations of *n* sampled lineages and **l** the vector of ancestral locations at the corresponding *n* –1 internal nodes of the phylogeny. The vector **t** consists of the 2n−1 (relative) ages associated to all nodes in the phylogeny. It is made of *n* observed values, corresponding to the dates at the tips, plus *n* –1 unknown dates of ancestral nodes that are estimated from the data. Hence, from a technical point of view, the *n* tip dates should be considered as data and **t** should refer to the dates at internal nodes only. In the present study **t** refers instead to the dates for the whole set of nodes to simplify the notation. *τ* is a ranked tree topology and *σ* the dispersal parameter governing the intensity with which lineage locations fluctuate during the course of evolution (i.e., along the phylogenetic tree (t,τ)) under the RRW model. Dispersal along each edge is thus governed by the product of the time elapsed along that edge, the value of *σ*, and that of an edge-specific relative dispersal parameter. Each of the *n* –1 relative dispersal parameters is distributed according to a gamma distribution with mean equal to 1.0 and SD set to 2.0. Moreover, relative dispersal rates are normalized following ref. 1 to avoid identifiability issues similar to those arising with relaxed clock models. h(t,τ|θ) is the joint density of a vector of node ages and the corresponding ranked tree topology conditioned on the (composite) generic parameter *θ* that governs the tree-generating process. For instance, in the case that the tree-generating process is Kingman’s coalescent (42), *θ* corresponds to the product of the effective population size by the generation time expressed in calendar units. Finally, let **a** be the alignment of *n* genetic sequences observed at the tip nodes of the tree. Additionally, we provide another component to the model, the spatial sampling density s(·). The underlying model here defines how the sites where sampling takes place are selected. **e** is the random variable corresponding to the vector of these *n* sampling locations.

### Spatial Sampling Schemes.

In the present work we consider that the spatial coordinates observed at the tips of the tree result from the combination of two stochastic processes. On the one hand, “lineage location” refers to the outcome of the spatial diffusion process, i.e., the stochastic process governing the evolution of the spatial coordinates of lineages along the phylogeny (with associated density f(·) and random variables $l^{*}$, **l**). On the other hand, “sampling sites” correspond to the spatial coordinates resulting from the sampling process (with associated density s(·) and random variable **e**). Furthermore, we distinguish two sampling schemes in the present study. Under the first one, referred to as the “detection scheme,” every case of an epidemic or every individual organism considered has the same chance to be sampled independently of its location in space and time. For example, one can consider the scenario where observers cover almost uniformly the whole geographical space and randomly detect, sample, and sequence the organism of interest. More specifically this could be the scenario of an epidemic that is monitored through a range of medical facilities almost uniformly covering a given area (typically, a country) and that sequence samples independently of their location. These conditions more likely apply to local epidemics than to large-scale epidemics, pandemics, or endemic infectious diseases.

The second sampling scheme, referred to as the “survey scheme,” is relevant to the situation where the organisms of interest are scattered throughout the whole habitat and samples are collected independently of the dynamics of the geographical spread of these organisms. This scheme matches with the situation occurring when a pandemic is no longer controlled and infected individuals are to be found all over the habitat. Samples are then collected at various, possibly arbitrary, points in space and time with the goal of characterizing a particular evolutionary or epidemiological feature of the pandemic. For the survey scheme, sampling is contingent on practical considerations (e.g., the financial cost of accessing a given area) rather than driven by the outcome of the evolutionary process as in the detection scheme.

### Statistical Modeling under the Two Sampling Schemes.

Bayesian parameter inference relies on the joint posterior density

[1] $$\begin{array}{l} p(l^{*},l,t,\tau ,\sigma ,\theta |a,e)=\frac{\mathrm{Pr}\;(a|\tau ,t)p(l^{*},l,t,\tau ,\sigma ,\theta ,e)}{p(a,e)}\\ \;\propto \mathrm{Pr}\;(a|\tau ,t)p(l^{*},l,t,\tau ,\sigma ,\theta ,e), \end{array}$$

where Pr (a|τ,t) is the probability of the sequence alignment given the phylogenetic tree, which is traditionally evaluated using Felsenstein’s pruning algorithm (43). The crux of the problem considered in this study lies in the term $p(l^{*},l,t,\tau ,\sigma ,\theta ,e)$. Under the detection scheme, sampling is performed conditioned on the outcome of the evolutionary process that generated $l^{*}$. Hence, the sampling sites **e** are fully determined by $l^{*}$. More specifically, the definition given to the density of sampling locations **e** conditioned on $l^{*}$ is

[2] $$s(e|l^{*},l,t,\tau ,\sigma ,\theta )=s(e|l^{*})≔\delta (e-l^{*}),$$

where δ(·) is the delta Dirac function. One then relies on the following expression for the joint posterior density of interest:

[3] $$\begin{array}{l} p(l^{*},l,t,\tau ,\sigma ,\theta |a,e)\\ \propto \mathrm{Pr}\;(a|\tau ,t)s(e|l^{*},l,t,\tau ,\sigma ,\theta )f(l^{*},l|t,\tau ,\sigma )h(t,\tau |\theta )\pi (\sigma ,\theta )\\ \propto \mathrm{Pr}\;(a|\tau ,t)f(l^{*},l|t,\tau ,\sigma )h(t,\tau |\theta )\pi (\sigma )\pi (\theta )\;\text{if}\;l^{*}=e, \end{array}$$

where $f(l^{*},l|t,\tau ,\sigma )$ corresponds to the Brownian diffusion model and is thereby given by the product of bivariate normal densities. h(t,τ|θ) is the density given by the tree-generating process, i.e., Kingman’s coalescent in our case. π(·) are prior densities. This expression is that put forward in ref. 22 and implemented in the popular Bayesian samplers BEAST (17) and BEAST2 (18).

Under the survey scheme, sampling is ignorant of the output of the evolutionary process governing the locations of lineages. As opposed to the detection scheme, the outcome of the evolutionary process at the sampled tips is “filtered” by the sampling sites. In other words, $l^{*}$ is conditioned on **e**. Hence, the modeling strategy followed here mirrors that used for the detection scheme, where **e** is conditioned on $l^{*}$ instead of the reverse for the survey scheme. The joint posterior density is then decomposed as

[4] $$\begin{array}{l} p(l^{*},l,t,\tau ,\sigma ,\theta |a,e)\\ \propto \mathrm{Pr}\;(a|\tau ,t)p(l^{*},l,t,\tau |\sigma ,\theta ,e)s(e)\pi (\sigma )\pi (\theta ), \end{array}$$

where s(e), the location sampling density, does not convey information about *θ* or *σ* in the present study. It would be possible to amend the current approach and use a homogeneous Poisson process to model the spatial sampling. One would then assume that the number of samples collected depends on the effective population size. The corresponding density, s(e|θ), would then play a role in the estimation of *θ*. A similar approach was implemented in ref. 33 to model the dependence between sampling intensity and effective population size when sequences are sampled serially through time.

The definition of the conditional density $p(l^{*},l,t,\tau |\sigma ,\theta ,e)$ is thus at the core of the survey scheme modeling approach. This density is null whenever $l^{*}$ differs from **e** and $p(l^{*},l,t,\tau |\sigma ,\theta ,e)\propto p(l^{*},l,t,\tau |\sigma ,\theta )$ when $l^{*}$ exactly matches **e**, so that we have

[5] $$p(l^{*},l,t,\tau |\sigma ,\theta ,e)=\{\begin{array}{ll} \frac{f(l^{*},l|t,\tau ,\sigma )h(t,\tau |\theta )}{Z(\sigma ,\theta )}, & \text{if}\;l^{*}=e\\ 0 & \text{otherwise,} \end{array}$$

where $Z(\sigma ,\theta )=f(l^{*}|\sigma ,\theta )=\sum_{\tau }\int f(l^{*}|t,\tau ,\sigma )h(t,\tau |\theta )\text{d}t$ is the probability density of all trees and internal node locations with $l^{*}=e$ as the vector of tip locations. Computing the value of Z(σ,θ) is challenging since it involves summing over all possible ranked tree topologies and, for each of them, integrating over all possible internal node ages. Because this term appears in the denominator in the expression above, the density $p(l^{*},l,t,\tau |\sigma ,\theta ,e)$ (considered here as a function of the dispersal and tree-generating parameters) is “flatter” than that of $f(l^{*},l|t,\tau ,\sigma )h(t,\tau |\theta )$, which is at the core of the detection scheme (Eq. **3**). This observation indicates that less information about the dispersal and tree-generating parameters is available under the survey scheme compared to the detection scheme, as one would expect.

Note that when *σ* increases, the population gets closer to panmixia. Z(σ,θ) becomes flatter and the inference of *θ* under the survey scheme is the same as that under the detection scheme, which amounts to the standard coalescent here. We verified that inference under a flat density for $f(l^{*},l|t,\tau ,\sigma )$ resulted indeed in identical posterior distributions for *θ* under both sampling schemes. When *σ* is very small, $f(l^{*},l|t,\tau ,\sigma )$ is sharply peaked around an optimal **l** and we have $Z(\sigma ,\theta )≃f(l^{*},l|t,\tau ,\sigma )h(t,\tau )$ so that $p(l^{*},l,t,\tau |\sigma ,\theta ,e)≃1$ and little information is available about *θ* under the survey scheme. Finally, when $f(l^{*}|\sigma ,\theta )\propto 1$, i.e., when the evolutionary process generates uniformly distributed locations at the tips of the reconstructed tree, then p(l,t,τ|σ,θ,e)=p(l,t,τ|σ,θ) and inference under both sampling schemes is equivalent.

### Bayesian Inference and the Exchange Algorithm.

In the context of Bayesian inference based on the Metropolis–Hastings (M-H) algorithm (44, 45), updating the value of the dispersal parameter (or that of the tree-generating model) under the survey scheme would involve the calculation of the acceptance probability $\alpha_{\sigma }$ defined as follows (we assume that $l^{*}=e$ in the following):

[6] $$\begin{array}{l} \alpha_{\sigma }=\min\left(1,\frac{p(l^{*},l,t,\tau ,\sigma \prime ,\theta |e)}{p(l^{*},l,t,\tau ,\sigma ,\theta |e)}\cdot \frac{q(\sigma |\sigma \prime )}{q(\sigma \prime |\sigma )}\right)\\ =\min\left(1,\frac{f(l^{*},l|t,\tau ,\sigma \prime )}{f(l^{*},l|t,\tau ,\sigma )}\cdot \frac{Z(\sigma ,\theta )}{Z(\sigma \prime ,\theta )}\cdot \frac{\pi (\sigma \prime )}{\pi (\sigma )}\cdot \frac{q(\sigma |\sigma \prime )}{q(\sigma \prime |\sigma )}\right). \end{array}$$

Calculating this probability is thus problematic since it relies on the ratio of normalizing terms Z(σ,θ)/Z(σ′,θ) and each of these two terms is computationally intractable. The same issue arises when updating the value of the parameter *θ*. Bayesian inference is thus here “doubly intractable”: Neither Z(σ,θ) nor p(a,e), i.e., the numerator in the Bayes formula (Eq. **1**), can be computed easily.

Fortunately, the exchange algorithm (37, 46) provides a way to generate correlated random draws from the target distribution that does not require evaluating any of the normalizing terms or ratios of these quantities. The technique described below is an extension of the original exchange algorithm described in ref. 38. The very same approach was used recently in the context of molecular dating in phylogenetics (47). Let $y≔(l^{*},l,t,\tau )$ be a composite random variable that includes the vectors of tip and internal node locations along with the phylogeny. Also, $x≔(l^{*},l,\;t,\psi )$ is an auxiliary random variable with structure similar to that of **y**. This composite random variable is made of vectors $l^{*}$ and l of *n* and *n* –1 spatial coordinates, respectively (with $l^{*}=e$); a vector t of 2n−1 node times; and *ψ*, a tree topology. In practice, when proposing new parameter values σ′ and θ′, x is sampled conditioned on σ′ and θ′ (see below) and is used to calculate the following acceptance probability (see *SI Appendix*, section 1 for details):

[7] $$\begin{array}{l} \alpha_{\sigma ,\theta }=\min\left(1,\frac{p(y,\sigma \prime ,\theta \prime ,x,\sigma ,\theta |e,a)}{p(y,\sigma ,\theta ,x,\sigma \prime ,\theta \prime |e,a)}\cdot \frac{q(\sigma ,\theta |\sigma \prime ,\theta \prime )}{q(\sigma \prime ,\theta \prime |\sigma ,\theta )}\right)\\ =\min(1,\frac{\pi (\sigma \prime )}{\pi (\sigma )}\cdot \frac{f(l^{*},l|t,\tau ,\sigma \prime )}{f(l^{*},l|t,\tau ,\sigma )}\cdot \frac{f(l^{*},l|t,\psi ,\sigma )}{f(l^{*},l|t,\psi ,\sigma \prime )}\cdot \\ \;\;\;\;\frac{h(t,\tau |\theta \prime )}{h(t,\tau |\theta )}\cdot \frac{h(t,\psi |\theta )}{h(t,\psi |\theta \prime )}\cdot \\ \;\;\;\;\frac{q(\sigma |\sigma \prime )}{q(\sigma \prime |\sigma )}\cdot \frac{q(\theta |\theta \prime )}{q(\theta \prime |\theta )}), \end{array}$$

where θ′ and σ′ are sampled from standard proposal distributions with densities q(·|σ) and q(·|θ), respectively.

Examination of the expression above first shows that the removal of all probability densities involving the auxiliary variable gives the corresponding acceptance ratio for the detection scheme. It also shows that proposed values of σ′ and θ′ that are poor with respect to **y**, thereby leading to a small Metropolis ratio (second and fourth ratios in Eq. **7**), may be offset by large Metropolis ratios involving the auxiliary variable .. (third and fifth ratios). One thus expects a higher posterior variance for *σ* and *θ* under the survey scheme as the auxiliary variable contributes to sampling more extreme values for these two parameters than one would do under the detection scheme.

Also, in the case that the signal conveyed by the sequences is weak, the following ratios of densities, p(y|σ′,θ′,e)/p(y|σ,θ,e) and p(x|σ,θ,e)/p(x|σ′,θ′,e), both have posterior expectations equal to 1 so that the posterior distributions of *θ* and *σ* are virtually identical to the prior. Therefore, under the survey scheme, the sampling locations do not convey direct information about these parameters. It is the conjunction of the phylogeny (informed by the sequence alignment) and the sampled locations that serves as a basis for the inference of the two parameters of interest. Under the detection scheme, the acceptance ratio for updating both *σ* and *θ* rests on the Metropolis ratio p(y|σ′,θ′)/p(y|σ,θ). The expectation of the latter is distinct from 1. Hence, one assumes here that the sampling locations mirror the outcome of the evolutionary processes and therefore convey information about the rate of dispersal (*SI Appendix*, section 2).

The computation of $\alpha_{\sigma ,\theta }$ in Eq. **7** does not involve any of the problematic normalizing terms seen above. The exchange algorithm thus provides an elegant approach for circumventing the computational challenge posed by this inference problem. Yet, this algorithm requires perfect sampling for x from the corresponding marginal distribution with density $f(l^{*},l|t,\psi ,\sigma \prime )h(t,\psi |\theta \prime )/Z(\sigma \prime ,\theta \prime )$, which is not feasible in our case. It is, however, possible to replace this step with a standard Metropolis–Hastings algorithm. A series of *m* M-H steps are thus used here to generate $x_{1},\cdots ,x_{m}$ with acceptance ratio for the *i*th step of this algorithm as follows:

[8] $$\begin{array}{l} \alpha_{x_{i}}=\min\left(1,\frac{p(x_{⋆}|e)}{p(x_{i}|e)}\cdot \frac{q(x_{i}|x_{⋆})}{q(x_{⋆}|x_{i})}\right)\\ =\min(1,\frac{f(l^{*},l_{⋆}|\;t_{⋆},\psi_{⋆},\sigma \prime )}{f(l^{*},l_{i}|t_{i},\psi_{i},\sigma \prime )}\cdot \frac{h(t_{⋆},\psi_{⋆}|\theta \prime )}{h(t_{i},\psi_{i}|\theta \prime )}\cdot \\ \;\;\;\;\frac{q(l_{i},t_{i},\psi_{i}|l_{⋆},t_{⋆},\psi_{⋆})}{q(l_{⋆},t_{⋆},\psi_{⋆}|l_{i},t_{i},\psi_{i})}), \end{array}$$

where symbols with a star (⋆) correspond to proposed values for x. The value of $x_{m}$ is then retained as a valid random draw from the target distribution, i.e., p(·|σ′,e) here. In practice, we used m=10n, where *n* is the number of sampled lineages. Larger values for this tuning parameter did not yield distinct parameter estimates.

Sampling of other model parameters in the phylogeographic model is conducted using standard operators that all rely on the Metropolis–Hastings algorithm. The operators implemented in this study (and available in the PhyREX software program) are similar to that employed by the BEAST sampler. Note, however, that BEAST relies on mathematical integration of the ancestral spatial locations given the observed ones and the phylogeny (39). In PhyREX, ancestral locations are explicit variables instead. Proposing sensible location values when updating the tree structure (through a “node slide” operator for instance) required the implementation of additional operators that are documented in the source code.

### Datasets.

We assessed the impact of sampling schemes through the analysis of real and simulated data. We first considered data from the recent WNV outbreak in North America (39). The corresponding alignment of georeferenced sequences is one of the “flagship” datasets used by the BEAST software package. A Hasegawa, Kishino, and Yano (HKY) substitution model (48), with nucleotide frequencies fixed to their empirical estimates and no rate variation across sites, was used for the sequence analysis. The molecular clock was calibrated using information that derived from the timing of collection of the various sequences (ranging from 1999 to 2007). Variation of substitution rates across edges in the phylogeny was modeled using a lognormal uncorrelated clock model, similar to that used by default in BEAST. Branch-specific substitution rates were normalized as in ref. 49. The tree-generating model was a Kingman coalescent with effective population size growing (or shrinking) exponentially (50). An exponential distribution with mean set to 10 was used as a prior for the effective population size parameter while a flat prior was applied to the exponential growth parameter. Finally, the evolution of the spatial coordinates along the phylogeny was modeled using the RRW model with dispersal along the east–west axis considered as independent of that along the north–south one. Here again, an exponential distribution with mean set to 10 was used as a prior for each of the two corresponding dispersal parameters.

We also simulated data to assess the impact of patterns in spatial sampling on the inference of dispersal parameters. Sequences and locations were generated following ref. 26. Trees with 1,000 tips were first synthesized under a Yule process with birth parameter set to 1.0. DNA sequences evolved along these trees under an HKY model with uniform nucleotide frequencies, a transition/transversion ratio was fixed to 3.0, and an average substitution rate was set to 0.01 substitution per base pair per time unit. Two independent Brownian processes then ran along the tree with both dispersal parameter values fixed to 1.0. The location at the root node was set to the point of coordinates (0,0). Seven sampling designs were considered. For design 1, 50 tips among the 1,000 tips from the full tree were selected uniformly at random. For design 2, the 50 tips with coordinates that are the closest from (0,0) were selected. For design 3, we selected the 50 tips that are the closest from the identity line. For design 4, the 50 tips with the highest longitudes were selected. For design 5, all 1,000 tips were given an exponential weight increasing with the longitude. Fifty tips were then randomly selected proportionally to these weights. For design 6, the 50 tips that were the closest from the points (–2,0) and (+2,0) were selected. For design 7, 50 tips were collected sequentially such that the distance between a newly selected tip and the previous ones is at least equal to 0.1. This last sampling design resulted in overdispersed samples compared to design 1. For each sampling design, 40 simulated datasets were analyzed using our Bayesian sampler under both sampling schemes. The length of the chain corresponding to each analysis was set to 1$\times 10^{8}$ steps.

## Supplementary Material

Supplementary File

## Data Availability

The Bayesian inference methods used in this study are implemented in the software program PhyREX, part of the PhyML package and available from GitHub, https://github.com/stephaneguindon/phyml. Instructions describing how to reproduce the analyses presented in this study can be found at GitHub, http://stephaneguindon.github.io/phylogeo.html. The West Nile virus dataset was downloaded from https://beast.community/workshop_continuous_diffusion_wnv. All study data are included in this article and/or *SI Appendix*. Previously published data were used for this work (https://journals.plos.org/ploscompbiol/article?id=10.1371/journal.pcbi.1008561).
