# Supplementary material for: Accounting for spatial sampling patterns in Bayesian phylogeography
Source: Proc Natl Acad Sci U S A. 2021 Dec 20;118(52):e2105273118. doi: 10.1073/pnas.2105273118 (PMC8719894; doi:10.1073/pnas.2105273118)
Supplement: Supplementary File [file pnas.2105273118.sapp.pdf]

PNAS
www.pnas.org

Supplementary Information for

Accounting for spatial sampling patterns in Bayesian phylogeography

Stéphane Guindon and Nicola De Maio

Stéphane Guindon.
E-mail: guindon@lirmm.fr

This PDF file includes:

Supplementary text
Figs. S1 to S4
Table S1

Supporting Information Text

1. Rationale behind the exchange algorithm

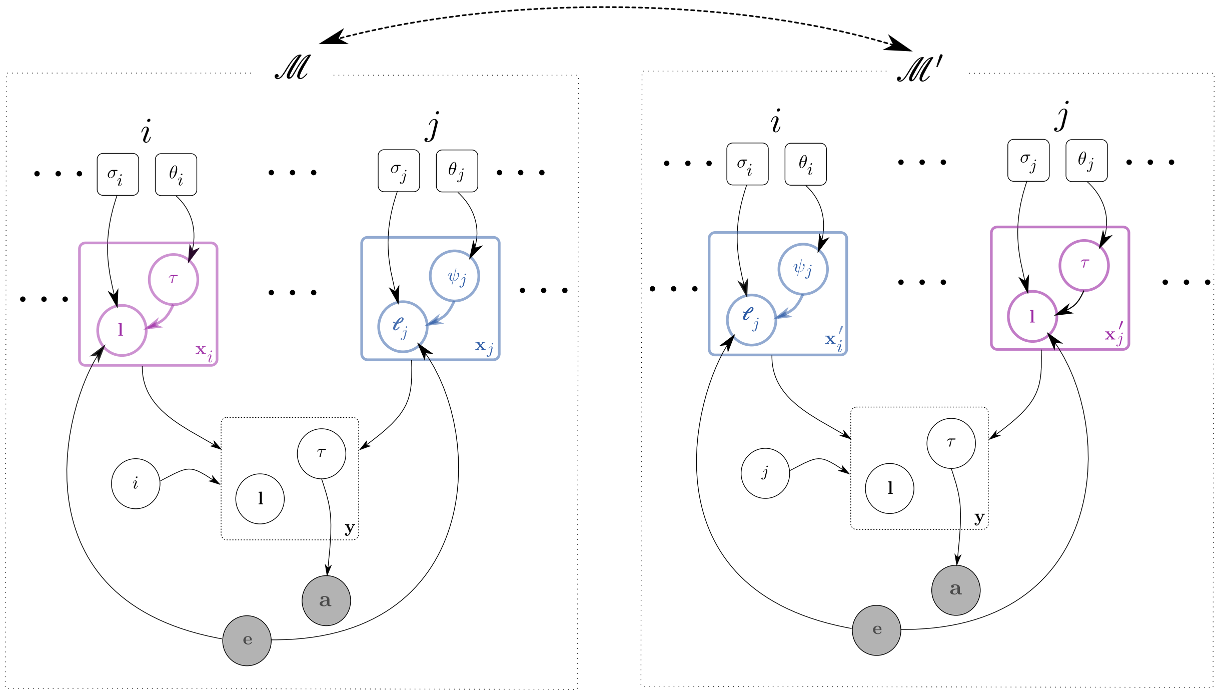

Fig. S1. Generative models used as a basis for the exchange algorithm. $\{\{\sigma_1, \theta_1\}, \ldots, \{\sigma_n, \theta_n\}\}$ are n fixed values for the dispersal and effective population size. i and j are values taken by a discrete random variable which distribution over $[1, n]$ is identical to the distribution over $\{\{\sigma_1, \theta_1\}, \ldots, \{\sigma_n, \theta_n\}\}$ (see text). This indicator is used to set $\mathbf{y} = \mathbf{x}_i$ (i.e., $\{\tau, \mathbf{t}, \mathbf{l}, \mathbf{l}^*\} = \{\psi_i, \boldsymbol{t}_i, \boldsymbol{\ell}_i, \boldsymbol{\ell}_i^*\}$) for the instance $\mathscr{M}$ of the model (left). The exchange $i \leftrightarrow j$ amounts to swapping $\mathbf{x}_i$ and $\mathbf{x}_j$ in $\mathscr{M}$, giving $\mathscr{M}'$ (right). For the sake of clarity, these graphs do not display the rate and substitution model parameters used to generate the sequence alignment $\mathbf{a}$ given the tree and node ages $(\tau, \mathbf{t})$.

The graphical model depicted in Figure S1 describes an approach that could be used to generate data under the survey sampling scheme. With this generative model, a series of parameter values $\{\{\sigma_1, \theta_1\}, \ldots, \{\sigma_n, \theta_n\}\}$, with n large, are fixed first (n is not to be confused here with the number of tips in the phylogeny), that is, we hereby consider a large discrete state space, as a way of approximating a continuous one. Each $\{\sigma_i, \theta_i\}$ then generates a corresponding pseudo tree $\{\psi_i, \boldsymbol{t}_i\}$ under the coalescent model, and pseudo locations $\{\boldsymbol{\ell}_i, \boldsymbol{\ell}_i^*\}$ under the spatial diffusion process, assuming that the survey scheme applies (i.e., conditioning on $\mathbf{e}$). Now, the discrete random variable i is an index value that has the same prior distribution as that of the joint prior of the dispersal and the effective population size parameters, i.e., $\Pr(i) = \Pr(\sigma_i, \theta_i)$. Moreover, we have $\Pr(\sigma_i, \theta_i) \propto \pi(\sigma_i) \cdot \pi(\theta_i)$ such that the probability of pairs of parameter values (σ_i, θ_i) as defined by the generative model are proportional to the corresponding prior probability densities under the inference model (i.e., the Brownian diffusion model with survey sampling scheme). The actual tree $\{\tau, \mathbf{t}\}$ and actual locations $\{\mathbf{l}, \mathbf{l}^*\}$ are then obtained by copying from $\{\psi_i, \boldsymbol{t}_i\}$ and $\{\boldsymbol{\ell}_i, \boldsymbol{\ell}_i^*\}$ respectively, i.e., $p(\mathbf{y}|\mathbf{x}_1, \ldots, \mathbf{x}_n, i) := \delta(\mathbf{y} - \mathbf{x}_i)$. Finally, genetic sequences are generated conditioned on $\{\tau, \mathbf{t}\}$ under standard models of substitution and rate variation along edges (not depicted in Figure S1 for the sake of clarity). The joint probability distribution (noted as $\mathscr{J}$) corresponding to this generative model is thus as follows:

$$\begin{aligned}
\mathscr{J} &= \Pr(\mathbf{a}|\mathbf{y}) \cdot \Pr(i) \cdot p(\mathbf{y}|\mathbf{x}_1, \ldots, \mathbf{x}_n, i) \cdot p(\mathbf{x}_1, \ldots, \mathbf{x}_n|\mathbf{e}, \sigma_1, \ldots, \sigma_n, \theta_1, \ldots, \theta_n) \\
&= \Pr(\mathbf{a}|\mathbf{y}) \cdot \Pr(\sigma_i, \theta_i) \cdot \delta(\mathbf{y} - \mathbf{x}_i) \cdot p(\mathbf{x}_1, \ldots, \mathbf{x}_n|\mathbf{e}, \sigma_1, \ldots, \sigma_n, \theta_1, \ldots, \theta_n) \\
&= \Pr(\mathbf{a}|\mathbf{y}) \cdot \Pr(\sigma_i, \theta_i) \cdot \delta(\mathbf{y} - \mathbf{x}_i) \cdot p(\mathbf{x}_i|\mathbf{e}, \sigma_i, \theta_i) \cdot \prod_{j \neq i} p(\mathbf{x}_j|\mathbf{e}, \sigma_j, \theta_j).
\end{aligned}$$

When marginalizing over $\{\mathbf{x}_1, \ldots, \mathbf{x}_n\}$, the previous density then becomes:

$$\begin{aligned}
\int \mathscr{J} \mathrm{d}(\mathbf{x}_1, \ldots, \mathbf{x}_n) &= \Pr(\mathbf{a}|\mathbf{y}) \cdot \Pr(\sigma_i, \theta_i) \cdot p(\mathbf{y}|\mathbf{e}, \sigma_i, \theta_i) \\
&\propto p(\sigma_i, \theta_i, \mathbf{y}|\mathbf{a}, \mathbf{e}),
\end{aligned}$$

thereby showing that the generative model depicted about is indeed a valid data generator under the model of interest.

Now, we consider different instances of this model as distinct states of a Markov chain (see Figure S1). We take a first instance of the generative model, noted as $\mathscr{M}$, whereby $\mathbf{x}_i = \mathbf{y}$ and a second instance, noted $\mathscr{M}'$, identical to $\mathscr{M}$ except for the

fact that $\mathscr{M}'$ has index value $j \neq i$ and for the fact that the values of $\mathbf{x}_i$ and $\mathbf{x}_j$ are swapped in $\mathscr{M}'$ (we have $\mathbf{x}'_i = \mathbf{x}_j$ and $\mathbf{x}'_j = \mathbf{x}_i = \mathbf{y}$). The ratio of joint probabilities below corresponds to the proposal $\mathscr{M}' \rightarrow \mathscr{M}$:

$$\begin{aligned}
\mathscr{W} = \frac{\mathscr{J}}{\mathscr{J}'} &= \frac{\Pr(\mathbf{a}|\mathbf{y}) \cdot \Pr(\sigma_i, \theta_i) \cdot \delta(\mathbf{y} - \mathbf{x}_i) \cdot p(\mathbf{x}_i|\mathbf{e}, \sigma_i, \theta_i) \cdot p(\mathbf{x}_j|\mathbf{e}, \sigma_j, \theta_j) \cdot \prod_{k \neq i,j} p(\mathbf{x}_k|\mathbf{e}, \sigma_k, \theta_k)}{\Pr(\mathbf{a}|\mathbf{y}) \cdot \Pr(\sigma_j, \theta_j) \cdot \delta(\mathbf{y} - \mathbf{x}'_j) \cdot p(\mathbf{x}'_i|\mathbf{e}, \sigma_i, \theta_i) \cdot p(\mathbf{x}'_j|\mathbf{e}, \sigma_j, \theta_j) \cdot \prod_{k \neq i,j} p(\mathbf{x}_k|\mathbf{e}, \sigma_k, \theta_k)} \\
&= \frac{\Pr(\sigma_i, \theta_i) \cdot \delta(\mathbf{y} - \mathbf{x}_i) \cdot p(\mathbf{x}_i|\mathbf{e}, \sigma_i, \theta_i) \cdot p(\mathbf{x}_j|\mathbf{e}, \sigma_j, \theta_j)}{\Pr(\sigma_j, \theta_j) \cdot \delta(\mathbf{y} - \mathbf{x}'_j) \cdot p(\mathbf{x}'_i|\mathbf{e}, \sigma_i, \theta_i) \cdot p(\mathbf{x}'_j|\mathbf{e}, \sigma_j, \theta_j)} \\
&= \frac{\Pr(\sigma_i, \theta_i) \cdot p(\mathbf{y}|\mathbf{e}, \sigma_i, \theta_i) \cdot p(\mathbf{x}_j|\mathbf{e}, \sigma_j, \theta_j)}{\Pr(\sigma_j, \theta_j) \cdot p(\mathbf{x}_j|\mathbf{e}, \sigma_i, \theta_i) \cdot p(\mathbf{y}|\mathbf{e}, \sigma_j, \theta_j)} \\
&= \frac{\pi(\sigma_i) \cdot \pi(\theta_i) \cdot f(\mathbf{y}|\sigma_i, \theta_i) \cdot f(\mathbf{x}_j|\sigma_j, \theta_j)}{\pi(\sigma_j) \cdot \pi(\theta_j) \cdot f(\mathbf{x}_j|\sigma_i, \theta_i) \cdot f(\mathbf{y}|\sigma_j, \theta_j)}.
\end{aligned} \quad [1]$$

It defines a valid Metropolis ratio for sampling from the joint posterior distribution of the dispersal and effective population size parameters. Importantly, the terms Z_{σ_i,θ_i} and Z_{σ_j,θ_j} cancel out in this ratio, making its computation feasible. These last expressions also show that the density that applies to $\mathbf{x}_i|\sigma_i, \theta_i$ has to match that of $\mathbf{y}|\sigma_i, \theta_i$, i.e., not any distribution involving Z_{σ_i,θ_i} in denominator would be suitable indeed here. The rationale behind the exchange algorithm is based on the same approach whereby the settings of $\mathbf{x}_i$ and $\mathbf{x}_j$ are swapped when updating the parameters $\{\sigma_j, \theta_j\} \rightarrow \{\sigma_i, \theta_i\}$ in a MCMC sampler.

Close examination of the last expression above sheds light on the rationale behind the role of the auxiliary random variable $\mathbf{x}$. Indeed, re-organizing Equation 10 (see main text), we have:

$$\mathscr{W} = \frac{\pi(\sigma_i) \cdot \pi(\theta_i) \cdot f(\mathbf{y}|\sigma_i, \theta_i)}{\pi(\sigma_j) \cdot \pi(\theta_j) \cdot f(\mathbf{y}|\sigma_j, \theta_j)} \cdot \frac{f(\mathbf{x}_j|\sigma_j, \theta_j)}{f(\mathbf{x}_j|\sigma_i, \theta_i)}, \quad [2]$$

which is to be compared with the acceptance ratio of a standard Metropolis-Hastings step for the update $(\sigma_j, \theta_j) \rightarrow (\sigma_i, \theta_i)$ (see Equation 6 in main text):

$$\alpha = \frac{\pi(\sigma_i) \cdot \pi(\theta_i) \cdot f(\mathbf{y}|\sigma_i, \theta_i)}{\pi(\sigma_j) \cdot \pi(\theta_j) \cdot f(\mathbf{y}|\sigma_j, \theta_j)} \cdot \frac{Z(\sigma_j, \theta_j)}{Z(\sigma_i, \theta_i)}. \quad [3]$$

The comparison of these last two expressions suggests a link between $\frac{f(\mathbf{x}_j|\sigma_j,\theta_j)}{f(\mathbf{x}_j|\sigma_i,\theta_i)}$ and $\frac{Z(\sigma_j,\theta_j)}{Z(\sigma_i,\theta_i)}$. This connection is further clarified by considering the expectation of $\frac{f(\mathbf{x}|\sigma_j,\theta_j)}{f(\mathbf{x}|\sigma_i,\theta_i)}$ over $\mathbf{x} \sim \frac{f(\cdot|\sigma_i,\theta_i)}{Z(\sigma_i,\theta_i)}$. We have indeed:

$$\begin{aligned}
\mathrm{E}\left(\frac{f(\mathbf{x}|\sigma_j, \theta_j)}{f(\mathbf{x}|\sigma_i, \theta_i)}\right) &= \int \frac{f(x|\sigma_j, \theta_j)}{f(x|\sigma_i, \theta_i)} \times \frac{f(x|\sigma_i, \theta_i)}{Z(\sigma_i, \theta_i)} \mathrm{d}x \\
&= \frac{1}{Z(\sigma_i, \theta_i)} \times \int f(x|\sigma_j, \theta_j) \mathrm{d}x \\
&= \frac{Z(\sigma_j, \theta_j)}{Z(\sigma_i, \theta_i)},
\end{aligned} \quad [4]$$

where the integral $\int f(x|\sigma_j, \theta_j)\mathrm{d}x = f(\mathbf{l}^*|\sigma_j, \theta_j) := Z(\sigma_j, \theta_j)$ is the probability density of all trees and internal node locations with $\mathbf{l}^* = \mathbf{e}$, in the context of interest here. Hence, the ratio $\frac{f(\mathbf{x}_j|\sigma_j,\theta_j)}{f(\mathbf{x}_j|\sigma_i,\theta_i)}$ is a one-step importance sampling approximation of $\frac{Z(\sigma_j,\theta_j)}{Z(\sigma_i,\theta_i)}$. In other words, the introduction of the auxiliary variable $\mathbf{x}$ permits the estimation of (the ratio of) the untractable normalization term $Z(\cdot)$ through a standard importance sampling scheme.

2. On the acceptance ratio under the survey scheme

The Metropolis ratio, noted as $\mathscr{W}$, at the core of the acceptance ratio for the exchange algorithm (see Eq. 7 in the main text) is as follows:

$$\mathscr{W} = \frac{p(\mathbf{y}|\sigma', \theta', \mathbf{e})}{p(\mathbf{y}|\sigma, \theta, \mathbf{e})} \cdot \frac{p(\mathbf{x}|\sigma, \theta, \mathbf{e})}{p(\mathbf{x}|\sigma', \theta', \mathbf{e})},$$

The posterior expectation of the ratio $p(\mathbf{y}|\sigma', \theta', \mathbf{e})/p(\mathbf{y}|\sigma, \theta, \mathbf{e})$ is then given as follows:

$$\mathrm{E}\left(\frac{p(\mathbf{y}|\sigma', \theta', \mathbf{e})}{p(\mathbf{y}|\sigma, \theta, \mathbf{e})}\right) = \int \frac{p(y|\sigma', \theta', \mathbf{e})}{p(y|\sigma, \theta, \mathbf{e})} p(y|\sigma, \theta, \mathbf{e}, \mathbf{a}) \mathrm{d}y.$$

If the signal conveyed about $\mathbf{y}$ by the sequence alignment is non-existent, then $p(\mathbf{y}|\sigma, \theta, \mathbf{e}, \mathbf{a}) = p(\mathbf{y}|\sigma, \theta, \mathbf{e})$ and the posterior expectation above becomes is then equal to 1.

Also, since $\mathbf{x}$ is a random draw from a distribution with density $p(\mathbf{x}|\sigma', \theta', \mathbf{e})$ (see graphical model in Supplementary Information 1), its posterior expectation is:

$$\mathrm{E}\left(\frac{p(\mathbf{x}|\sigma, \theta, \mathbf{e})}{p(\mathbf{x}|\sigma', \theta', \mathbf{e})}\right) = \int \frac{p(x|\sigma, \theta, \mathbf{e})}{p(x|\sigma', \theta', \mathbf{e})} p(x|\sigma', \theta', \mathbf{e})\mathrm{d}x = 1.$$

Altogether, we thus have $\mathrm{E}(\mathscr{W}) = \mathrm{E}\left(\frac{p(\mathbf{y}|\sigma', \theta', \mathbf{e})}{p(\mathbf{y}|\sigma, \theta, \mathbf{e})}\right) \cdot \mathrm{E}\left(\frac{p(\mathbf{x}|\sigma, \theta, \mathbf{e})}{p(\mathbf{x}|\sigma', \theta', \mathbf{e})}\right) = 1$ which establishes that, when taken alone, $\mathbf{e}$ does not convey any signal about θ and σ.

Under the detection scheme, the corresponding Metropolis ratio is as follows:

$$\mathscr{W} = \frac{p(\mathbf{y}|\sigma', \theta')}{p(\mathbf{y}|\sigma, \theta)}.$$

When considering that $\mathbf{a}$ does not convey any signal, we have $p(\mathbf{y}|\sigma, \theta, \mathbf{e}, \mathbf{a}) = p(\mathbf{y}|\sigma, \theta, \mathbf{e})$ so that the posterior expectation of the Metropolis ratio is:

$$\begin{aligned}
\mathrm{E}\left(\frac{p(\mathbf{y}|\sigma', \theta', \mathbf{e})}{p(\mathbf{y}|\sigma, \theta, \mathbf{e})}\right) &= \int \frac{p(y|\sigma', \theta')}{p(y|\sigma, \theta)} p(y|\sigma, \theta, \mathbf{e}, \mathbf{a})\mathrm{d}y \\
&= \int \frac{p(y|\sigma', \theta')}{p(y|\sigma, \theta)} p(y|\sigma, \theta, \mathbf{e})\mathrm{d}y \\
&\neq 1,
\end{aligned}$$

thereby showing that the detection scheme relies on the assumption that the sampling locations $\mathbf{e}$ convey information about the values of σ and θ.

3. Sensitivity analysis for the WNV data set

Figure S2 presents the posterior distributions of θ and σ under the survey and detection schemes for various prior settings on the two parameters.

4. Additional simulation results

Figures S3 and S4 display the posterior distributions of the longitudinal and latitudinal components of σ for seven sampling designs under the two sampling scheme.

Table S1 gives the precision and accuracy of the inference under both sampling scheme for the simulated when using a prior mean on the dispersal parameter set to 2.0 instead of 10.0 (see main text).

Sampling scheme / Sampling design			detection		survey	
			$[q_{0.025}, q_{0.975}]$	% correct	$[q_{0.025}, q_{0.975}]$	% correct
(i)	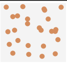	Lat.	$[0.74, 1.67]$	0.95	$[0.90, 2.87]$	0.68
		Lon.	$[0.78, 1.73]$	0.88	$[0.90.2.80]$	0.78
(ii)	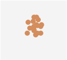		$[0.11, 0.21]$	0	$[0.18, 1.17]$	0.35
			$[0.11, 0.23]$	0	$[0.23, 1.57]$	0.48
(iii)	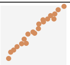		$[0.38, 0.85]$	0.20	$[0.56, 4.47]$	1
			$[0.38, 0.85]$	0.18	$[0.62, 4.80]$	0.93
(iv)	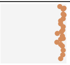		$[0.73, 1.63]$	0.98	$[0.78, 2.24]$	0.95
			$[0.29, 0.69]$	0.18	$[0.48, 2.45]$	0.90
(v)	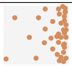		$[0.75, 1.71]$	0.93	$[0.87, 2.74]$	0.78
			$[0.54, 1.22]$	0.73	$[0.63, 2.13]$	0.98
(vi)	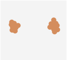		$[0.10, 0.17]$	0	$[0.17, 0.96]$	0.28
			$[0.45, 1.02]$	0.50	$[0.93, 3.45]$	0.70
(vii)	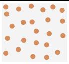		$[0.74, 1.65]$	0.88	$[0.91, 3.06]$	0.70
			$[0.76, 1.70]$	0.90	$[0.91, 2.93]$	0.73

Table S1. Accuracy and precision of dispersal rate estimates under seven spatial sampling designs: comparison of the detection and survey schemes. See caption of Table 1 in the main text. The mean prior values of the dispersal parameters was set to 2 instead of 10 as for the results presented in the main text.

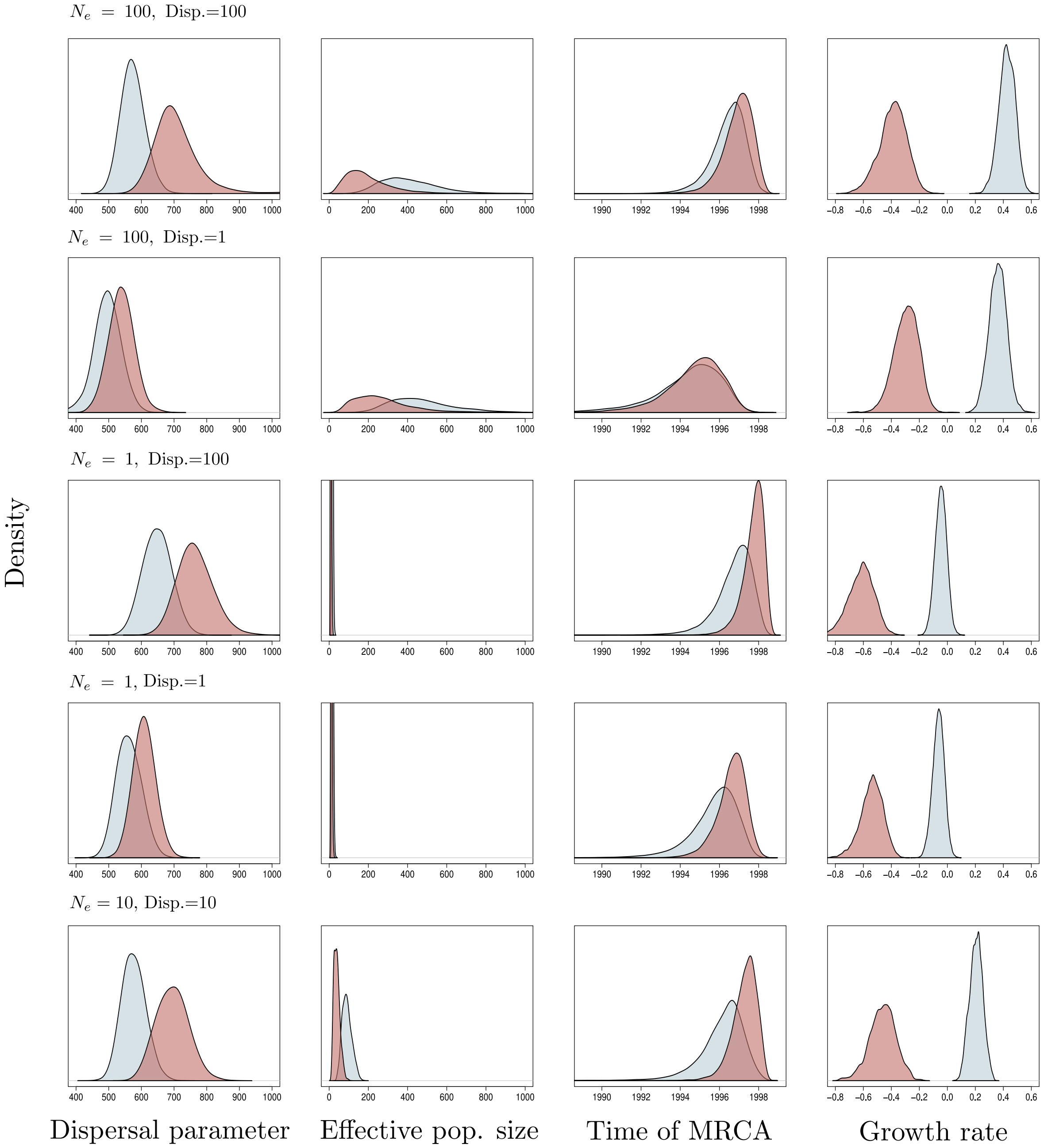

Fig. S2. Posterior distributions of relevant parameters for various prior distribution settings on the effective population size and the diffusion parameters. Values of 'N_e' and 'Disp.' above each row of four plots correspond to the mean of the exponential distribution that serves as prior for the effective population size parameter and the two dispersal parameters respectively.

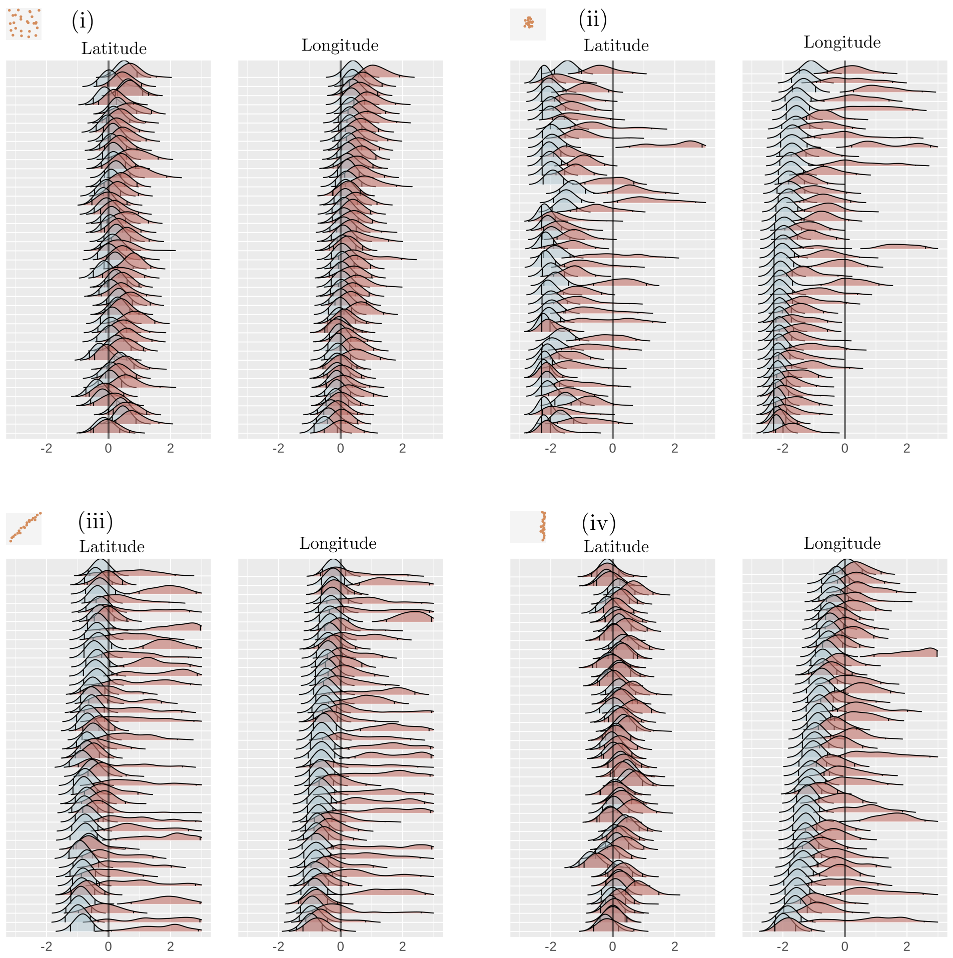

Fig. S3. Posterior distributions of the logarithm of dispersal parameters estimated from data simulated under distinct sampling designs. For each sampling design (from (i) to (iv)), 40 data sets were analyzed. The red distributions were obtained under the survey scheme. The blue ones were obtained under the detection scheme. Each data set was simulated using a value of the dispersal parameter along the East-West (longitude) axis equal to 1.0. The dispersal parameter along the North-South axis (latitude) was also set to 1.0 when generating each data set.

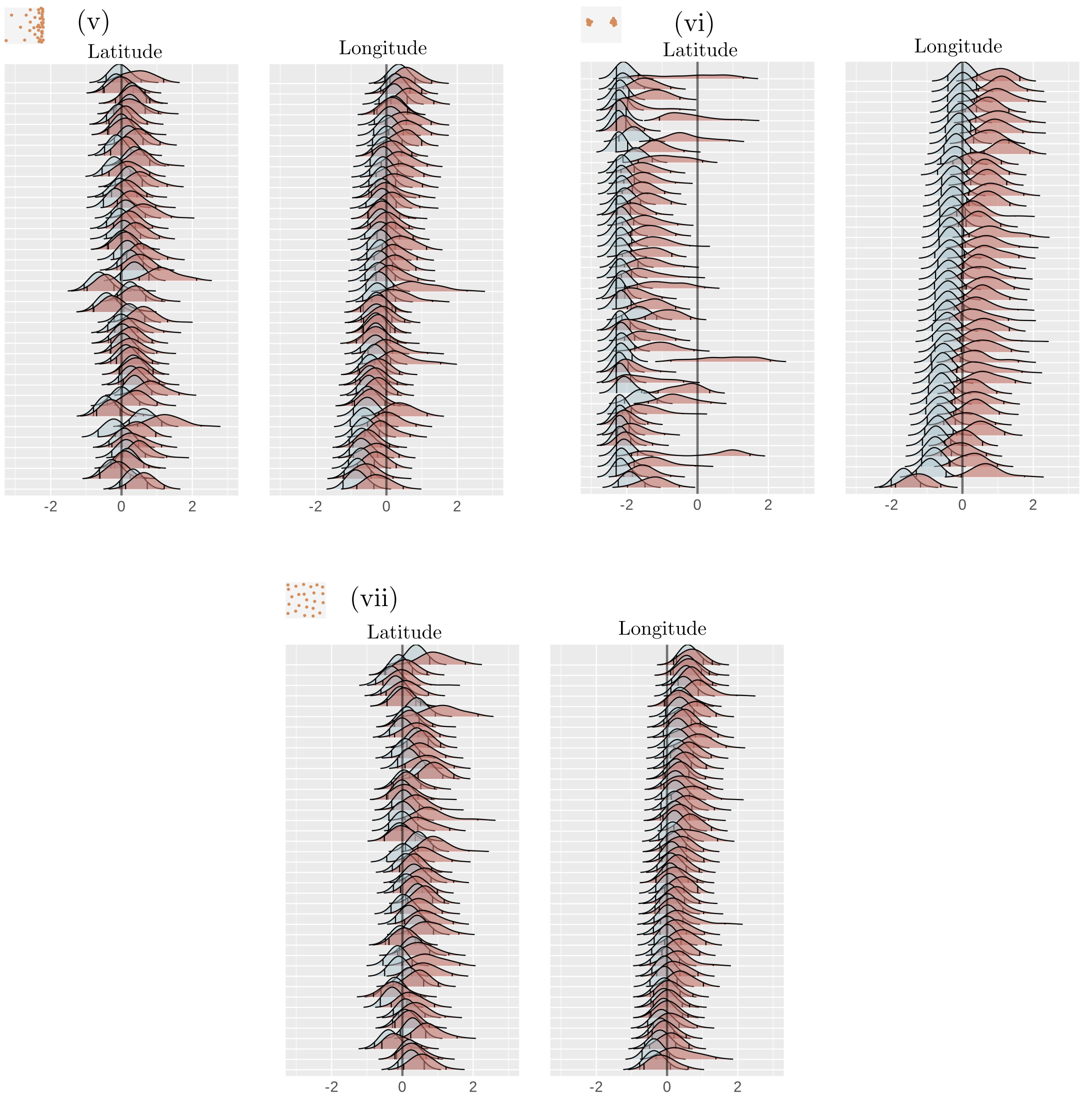

Fig. S4. Posterior distributions of the logarithm of dispersal parameters estimated from data simulated under distinct sampling designs (v) to (vii). See caption from previous figure.
